# The effectiveness of the hemoglobin, albumin, lymphocyte, and platelet (HALP) score in predicting lymph node metastasis in radiologically n0 locally advanced upper rectal cancer

**DOI:** 10.3389/fonc.2025.1579581

**Published:** 2025-07-04

**Authors:** Osman Bardakçı, Gökay Çetinkaya

**Affiliations:** ^1^ Department of Surgical Oncology, Isparta City Hospital, Isparta, Türkiye; ^2^ Department of Surgical Oncology, Ankara Gülhane Research and Training Hospital, Ankara, Türkiye

**Keywords:** HALP score, locally advanced upper rectal cancer, imaging, lymph node metastasis, surgery

## Abstract

**Background and Objective:**

Reliable preoperative identification of lymph node metastasis in clinically node-negative (cN0) patients with locally advanced upper rectal cancer (LAURC) remains a major clinical challenge due to the limited sensitivity of imaging modalities. The HALP score—calculated from hemoglobin, albumin, lymphocyte, and platelet counts—has emerged as a promising marker reflecting systemic inflammation and nutritional status. This study aimed to investigate the association between preoperative HALP scores and histopathologically confirmed lymph node metastasis in cN0 LAURC patients and to assess its diagnostic performance.

**Methods:**

This retrospective study included 62 patients who underwent curative resection for cN0 LAURC between January 2020 and December 2023. HALP scores were computed using the formula: hemoglobin (g/L) × albumin (g/L) × lymphocyte count (/L) ÷ platelet count (/L), based on fasting blood samples collected within one week prior to surgery. Patients were stratified according to the presence or absence of pathological lymph node metastasis. ROC curve analysis was used to determine the optimal HALP cut-off value. Strict exclusion criteria were applied to minimize confounding from comorbidities affecting hematologic parameters.

**Results:**

Lymph node metastasis was confirmed in 21 patients (33.9%). Patients with metastasis had significantly lower HALP scores compared to those without (p = 0.007). ROC analysis identified a HALP cut-off value of 6.98, yielding a sensitivity of 73% and specificity of 81% (AUC = 0.695; 95% CI: 0.56–0.83; p = 0.013). No significant associations were observed between HALP score and TNM stage or demographic variables.

**Conclusion:**

The HALP score is significantly associated with pathological lymph node metastasis in cN0 LAURC patients and may serve as a simple, inexpensive, and clinically applicable biomarker to support preoperative staging. Further prospective studies with survival-based endpoints are warranted to validate its prognostic value.

## 1 Introduction

Colorectal cancer (CRC) remains one of the most common malignancies globally and a leading contributor to cancer-related mortality. Approximately one-third of CRC cases involve the rectum, and among these, upper rectal cancers (URCs) constitute a clinically significant subgroup, accounting for 30–49% of all rectal tumors (1). Locally advanced rectal cancer (LARC), characterized by tumor invasion beyond the muscularis propria—classified as T3 when extending into perirectal tissues and T4 when involving adjacent organs—presents distinct therapeutic challenges. Lymph node metastasis, a hallmark of aggressive tumor biology, is observed in 18–24% of T2 tumors and increases in frequency with advancing T stage (2). Its presence is strongly associated with poorer oncological outcomes and informs the need for adjuvant therapy.

While total mesorectal excision (TME) following neoadjuvant chemoradiotherapy represents the standard of care in mid and lower rectal cancers, treatment strategies for locally advanced upper rectal cancer (LAURC) remain controversial (3). There is ongoing debate regarding the necessity and extent of mesorectal excision—partial versus total—and whether surgery alone suffices in the absence of clinically evident nodal disease. Despite its generally favorable prognosis compared to distal rectal cancers, LAURC demonstrates recurrence rates ranging from 5.7% to 44.6%, underlining the need for improved risk stratification tools (4–6).

Preoperative imaging modalities, particularly computed tomography (CT) and magnetic resonance imaging (MRI), are routinely employed to evaluate tumor extent and nodal involvement. However, their sensitivity and specificity in detecting microscopic lymph node metastases remain suboptimal. Reported accuracy rates for CT in determining tumor depth and nodal status range from 50% to 70% and 56% to 84%, respectively (7). MRI offers modestly improved performance, with accuracy rates of up to 75% for local invasion and 59% to 83% for lymph node evaluation (8–10). These limitations highlight the need for complementary, cost-effective biomarkers that can enhance preoperative staging accuracy, particularly in cN0 patients.

The Hemoglobin, Albumin, Lymphocyte, and Platelet (HALP) score has recently gained attention as a composite biomarker reflecting systemic inflammation and nutritional status—two critical dimensions of cancer progression. First introduced in 2016 in the context of gastrointestinal cancers, the HALP score has demonstrated prognostic value for overall and disease-free survival in CRC and other solid tumors (11–16). Its utility is reinforced by its derivation from routine hematologic parameters, making it an accessible and reproducible tool in the clinical setting.

To date, no study has specifically evaluated the HALP score’s capacity to predict pathological lymph node metastasis in patients with LAURC staged as clinically node-negative (cN0) by imaging. This study addresses this knowledge gap by investigating the association between preoperative HALP scores and histologically confirmed nodal metastases in cN0 LAURC patients. We hypothesize that lower HALP scores are associated with a higher likelihood of occult nodal involvement, and that this biomarker may support more personalized treatment planning in this patient subgroup.

## 2 Materials and methods

### 2.1 Study design and patient selection

This retrospective, single-center, cross-sectional study was conducted at the Department of Surgical Oncology and General Surgery, Isparta City Hospital, between January 2020 and December 2023. A total of 62 patients with histologically confirmed, clinically node-negative (cN0), locally advanced upper rectal cancer (LAURC) classified as T3 or T4 stage were included. Clinical staging was based on preoperative imaging, including contrast-enhanced computed tomography (CT), pelvic magnetic resonance imaging (MRI), and proctosigmoidoscopy, in accordance with international colorectal cancer management guidelines.

Eligible patients met the following criteria: (1) cN0 status on imaging, (2) surgical management via open or laparoscopic resection with mesorectal excision, (3) absence of prior neoadjuvant treatment, and (4) availability of complete preoperative laboratory and postoperative histopathological data. Exclusion criteria included patients with lower or mid-rectal cancer, T1–T2 tumors, radiologically suspicious lymph node involvement, distant metastases, benign pathology, or incomplete clinical data. This rigorous selection process ensured a clinically homogeneous and representative study population.

To assess the statistical adequacy of the sample size, a *post hoc* power analysis was conducted. Based on HALP score differences between patients with (n = 21) and without (n = 41) lymph node metastasis, an effect size of 0.77 was observed, yielding a statistical power of 87% at an alpha level of 0.05. This confirmed that the sample size was sufficient to detect meaningful differences.

### 2.2 Data collection and standardization

Demographic and clinical data—including age, sex, radiological findings, surgical technique, histopathological staging, and length of hospital stay—were retrieved from the institutional electronic health records. All patients underwent preoperative blood sampling within seven days prior to surgery, following overnight fasting, during standardized morning hours to ensure procedural uniformity.

To minimize the influence of confounding factors on hematologic parameters, patients with chronic inflammatory diseases, autoimmune disorders, active infections, hematologic malignancies, chronic liver disease, thrombocytopenia, or thrombocytosis were excluded. Platelet values and other hematologic indices were verified to fall within normal reference ranges prior to HALP calculation.

### 2.3 HALP score calculation

The HALP score was calculated using the following validated formula:HALP = Hemoglobin (g/L) × Albumin (g/L) × Lymphocyte count (×10^9^/L) ÷ Platelet count (×10^9^/L).Laboratory values were obtained directly from the institutional electronic database. To eliminate inter-observer variability and ensure consistency, HALP scores were computed using a predefined, automated spreadsheet formula applied uniformly across all cases.

### 2.4 Statistical analysis

All statistical analyses were performed using IBM SPSS Statistics for Windows, version 20.0 (IBM Corp., Armonk, NY, USA). The Kolmogorov–Smirnov test was used to assess the normality of continuous variables. For normally distributed variables, comparisons between groups were conducted using the Independent Samples t-test, while the Mann–Whitney U test and Kruskal–Wallis test were employed for non-normally distributed data. Receiver operating characteristic (ROC) curve analysis was utilized to evaluate the diagnostic performance of the HALP score in predicting lymph node metastasis. The optimal HALP cut-off value was determined using the Youden index. The area under the curve (AUC), sensitivity, specificity, and 95% confidence intervals (CIs) were reported. A two-tailed p-value < 0.05 was considered statistically significant for all tests.

## 3 Results

In this retrospective analysis of 62 patients with clinically node-negative (cN0) locally advanced upper rectal cancer (LAURC), the relationship between preoperative HALP score and postoperative lymph node metastasis was systematically evaluated. The mean age of the cohort was 68.48 ± 12.34 years (range: 45–95), with a slight male predominance (n = 33; 53.2%). All patients underwent surgery without prior neoadjuvant treatment. Based on pathological staging, 53 patients (85.5%) were classified as T3, while 9 patients (14.5%) were T4. Lymph node metastasis was identified in 21 patients (33.9%).

Regarding surgical approach, 17 patients (27.4%) underwent open low anterior resection with partial mesorectal excision, whereas 45 patients (72.6%) were managed laparoscopically. Postoperative complications were observed in 8 cases (13%), with wound infection being the most common. The mean number of lymph nodes harvested was 17 ± 7.21 (range: 5–39), and adequate lymphadenectomy (≥12 nodes) was achieved in 55 patients (85.7%). Preoperative hematologic parameters were as follows: hemoglobin 11.75 g/dL (range: 7.8–16.4), albumin 3.25 g/dL (range: 1.80–4.90), lymphocyte count 1.37 ×10³/μL (range: 0.21–4.79), and platelet count 269.52 ×10³/μL (range: 138–559) (**Table 1**).

**Table 1:** General characteristics of the patients.

Age, year, mean ± SD, distribution	68,48 ± 12,34 (45-95)
Age, n(%)	
Male	33 (53.2%)
Female	29 (46.8%)
Hgb, g/dL, mean ± SD, distribution	11.75 ± 1.8 (7.8-16.4)
Albumin, g/dL, mean ± SD, distribution	3.25 ± 0.63 (1.80-4.90)
Lymphocyte, 103/μl, mean ± SD, distribution	1.37 ± 0.77 (0.21-4.79)
Platelet, 103/μl, mean ± SD, distribution	296.52 ± 103.86 (138-559)
Number of lymph nodes, mean ± SD, distribution	17 ± 7.21 (5-39)
HALP Score, mean ± SD, distribution	19.61 ± 13.06 (3.54-59)
Lymph node metastasis, n(%)	
None	41 (66.12%)
There is	21 (33.88%)
Degree of T invasion, n(%)	
T3	53 (85.48%)
T4	9 (14.52%)
N stage grade, n(%)	
N0	41 (66.12%)
N1	14 (22.58%)
N2	7 (11.29%)
Hospitalization, days, mean ± SD, distribution	9.42 ± 2,87

SD, standard deviation; Hgb, Hemoglobin.

No significant association was found between HALP scores and patient sex or tumor T/N stage (p > 0.05). However, patients with lymph node metastasis exhibited significantly lower HALP scores compared to node-negative individuals (p = 0.007), indicating a potential diagnostic utility (**Table 2**).

**Table 2:** Distribution of clinico-pathological factors according to HALP scoring system.

Clinico-pathological factors	HALP score, median, range	P value
Gender, n(%)		
Male	20.15 (4.48-47.9)	p=0.731^U^
Female	19 (3.54-55.46)	
Degree of T invasion		
T_3_	32.04	P=0.527^U^
T_4_	27.29	
N Stage grade		
N_0_	33.04	P=0.272^K^
N_1_	29.73	
N_2_	18.25	
Lymph node metastasis		
No	22.38 (3.54-59)	**P=0.007^U^**
Yes	14.22 (3.6-34.19)	

U, Mann Whitney U test.

K, Kruskal-Wallis Test. Bold values indicate statistically significant differences.

**Figure 1:**
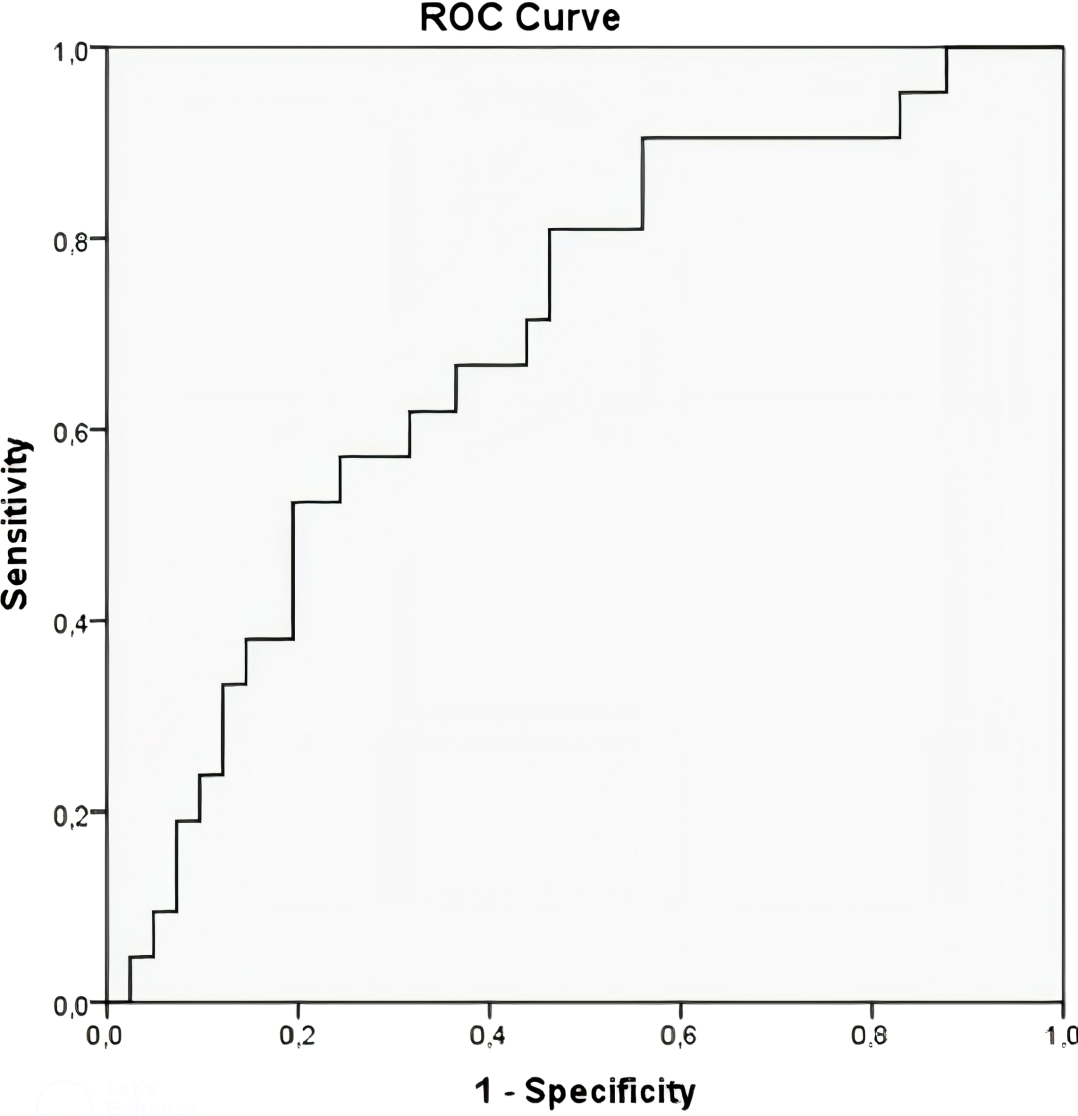
HALP score ROC curve in predicting lymph node metastasis.

Receiver operating characteristic (ROC) curve analysis was employed to assess the discriminatory performance of the HALP score for predicting lymph node metastasis. The optimal cut-off value was calculated as 6.98 based on the Youden index, yielding a sensitivity of 73% and specificity of 81%. The area under the ROC curve (AUC) was 0.695 (95% CI: 0.56–0.83), with a p-value of 0.013, indicating moderate predictive ability and statistical significance (**Table 3**; **Figure 1**).

**Table 3:** HALP score cut-off value in predicting lymph node metastasis.

	AUC (95% CI)	Cut-off	Sensitivity (%)	Specificity (%)	p value
Lymph node metastasis	0.695 (0.56-0.83)	6.98	73	81	0.013

AUC, Area under the curve; CI, Confidence interval.

## 4 Discussion

Accurate preoperative staging remains a cornerstone in the optimal management of rectal cancer (RC), as lymph node involvement is a critical determinant of prognosis, recurrence risk, and treatment planning (17, 18). Although magnetic resonance imaging (MRI) is considered the most reliable imaging modality and is widely recommended in colorectal cancer (CRC) staging guidelines (19), its diagnostic accuracy is notably reduced in upper rectal cancers (URCs) due to bowel loop interference and anatomical challenges (20). Computed tomography (CT), while frequently used, is limited by low contrast resolution and suboptimal sensitivity (55%) and specificity (74%) for detecting lymph node metastases, which are only marginally improved in MRI (66% and 76%, respectively) (21). These constraints underscore the need for complementary, accessible biomarkers that can enhance staging precision.

In recent years, systemic inflammatory and nutritional indices have gained attention for their prognostic significance in cancer patients. The Hemoglobin, Albumin, Lymphocyte, and Platelet (HALP) score, a composite biomarker derived from routine laboratory parameters, reflects both inflammatory status and nutritional reserves. Low albumin and hemoglobin levels are well-documented indicators of poor prognosis in gastrointestinal malignancies (22–24). Lymphocytes are key mediators of tumor immune surveillance, and lymphopenia has been correlated with worse oncologic outcomes (25). Platelets promote tumor angiogenesis and immune evasion via vascular endothelial growth factor (VEGF) and other pro-inflammatory mediators (26–29). By integrating these four markers, the HALP score provides a consolidated and clinically practical assessment of host-tumor interactions (30, 31).

In this study, we demonstrated that cN0 LAURC patients with a HALP score ≤6.98 had a significantly higher risk of pathological lymph node metastasis. These findings suggest that the HALP score can serve as a surrogate marker for occult nodal disease, even in the absence of radiological suspicion. Prior research has validated the prognostic utility of the HALP score in multiple malignancies. Jiang et al. (16) first introduced the HALP score in 2016 as a predictor of overall and disease-specific survival in patients with locally advanced CRC. Yalav et al. (32) associated low HALP scores with elevated carcinoembryonic antigen (CEA) levels and mucinous histology in CRC. Akbaş et al. (33) suggested its role in differentiating malignant and benign causes of intestinal obstruction, while Topal et al. (34) linked HALP scores to tumor budding—an indicator of aggressive tumor biology. In gastric cancer, Wang et al. (35) reported that patients with low HALP scores were four times more likely to present with nodal metastases.

The current study builds on these findings by exploring the HALP score specifically in the context of clinically node-negative, locally advanced upper rectal cancer—a population for which no previous HALP-based nodal predictive studies exist. This is especially relevant given the limited accuracy of CT and MRI in detecting microscopic nodal involvement (19–21), and it offers a valuable, cost-effective adjunct to conventional imaging.

## 5 Limitations and future directions

Several limitations must be acknowledged. First, the study’s retrospective and single-center design may limit generalizability and introduce selection bias. Second, the modest sample size, particularly in the T4 subgroup, may reduce the statistical power for detecting smaller effects. Third, due to the retrospective nature of data collection, survival outcomes such as overall survival (OS) and disease-free survival (DFS) could not be assessed, precluding Kaplan–Meier or Cox regression analyses.

Despite these limitations, the study’s principal strength lies in its originality. To the best of our knowledge, this is the first study to evaluate the predictive performance of the HALP score for lymph node metastasis in cN0 LAURC patients. Future multicenter, prospective studies with larger cohorts and long-term follow-up are warranted to validate our findings. Incorporating HALP into multivariate prognostic models alongside imaging and molecular markers could enhance its clinical applicability in personalized treatment planning.

## 5 Conclusions

The HALP score, derived from readily available preoperative laboratory parameters, demonstrates significant predictive value for pathological lymph node involvement in clinically node-negative locally advanced upper rectal cancer. A HALP threshold ≤6.98 was significantly associated with nodal positivity, highlighting its potential as a low-cost, non-invasive adjunct to conventional imaging. These findings support the integration of HALP into preoperative assessment protocols, with the aim of improving risk stratification and guiding individualized management strategies in rectal cancer.

## Data Availability

The raw data supporting the conclusions of this article will be made available by the authors, without undue reservation.
